# Robotic Rectus Muscle Flap Reconstruction After Pelvic Exenteration in Gynecological Oncology: Current and Future Perspectives—A Narrative Review

**DOI:** 10.3390/cancers18030375

**Published:** 2026-01-25

**Authors:** Gurhan Guney, Ritchie M. Delara, Johnny Yi, Evrim Erdemoglu, Kristina A. Butler

**Affiliations:** Department of Medical and Gynecologic Surgery, Mayo Clinic, Phoenix, AZ 85054, USA; guney.gurhan@mayo.edu (G.G.); delara.ritchie@mayo.edu (R.M.D.); yi.johnny@mayo.edu (J.Y.); erdemoglu.evrim@mayo.edu (E.E.)

**Keywords:** robotic rectus flap, pelvic exenteration, gynecologic oncology, reconstructive surgery, minimally invasive techniques

## Abstract

**Background/Objectives**: Pelvic exenteration is a radical procedure performed for recurrent gynecologic cancers. The goal of exenteration is to prolong survival, but this procedure also results in extensive tissue loss and consequently high morbidity. Reconstruction using vascularized flaps, particularly the VRAM flap, is crucial to restoring pelvic integrity and decreasing complications resulting from extensive tissue loss. With the rise of minimally invasive surgery, the traditionally open abdominal approach to exenteration and reconstruction can now be performed with the assistance of robotic platforms. This review aims to summarize available evidence, describe techniques, and propose future directions for robotic rectus flap reconstruction after pelvic exenteration. **Methods**: This narrative review was conducted following the SANRA guidelines for narrative synthesis. A comprehensive search of PubMed, Embase, Scopus, and Web of Science was conducted for studies published between January 2000 and November 2025 on pelvic exenteration followed by robotic rectus abdominis flap reconstruction in gynecologic oncology. Eligible studies were retrospective or prospective reports, technical descriptions, case series, or comparative analyses. Non-robotic techniques and animal studies were excluded. Although the primary focus was gynecologic oncology, technically relevant studies from other oncologic disciplines were included when the reconstructive approach was directly applicable to pelvic exenteration. Extracted data included patient demographics, surgical details, and perioperative and oncologic outcomes. **Results**: The literature search identified primarily case reports and small single-center series describing robot-assisted rectus muscle-based flap reconstruction after pelvic exenteration. Reported cases demonstrated technical feasibility and successful flap harvest using robotic platforms, with adequate pelvic defect coverage. Potential benefits, such as reduced wound morbidity and preservation of a minimally invasive workflow, have been described. However, patient numbers were small, techniques varied, and standardized outcome measures or comparative data with open approaches were lacking. **Conclusions**: Robotic rectus flap reconstruction represents a promising advancement in pelvic exenteration surgery, potentially reducing morbidity and improving recovery. Further research, including multicenter prospective studies, is needed to validate these findings and establish standardized protocols.

## Simple Summary

Pelvic exenteration is a radical procedure performed for recurrent or locally advanced gynecologic cancers and requires advanced anatomical knowledge. While extensive tissue loss during this procedure can lead to high morbidity, the procedure offers long-term survival potential. Reconstruction using vascularized flaps, such as the vertical rectus abdominis musculocutaneous (VRAM) or the transverse rectus abdominis musculocutaneous (TRAM) flap, has become the gold standard in open surgery for restoring pelvic integrity, reducing complications, and improving quality of life. Following introduction of minimally invasive pelvic exenteration, the need for minimally invasive reconstructive surgery has emerged. Current data on robotic rectus flap reconstruction is insufficient and limited to case reports and small series. This review analyzes the current literature, summarizes surgical techniques, and highlights future research needs to guide the integration of robot-assisted rectus muscle-based flap reconstruction into modern reconstructive algorithms.

## 1. Introduction

Pelvic exenteration is a radical oncologic surgery involving partial or total resection of pelvic organs, including reproductive structures, the bladder, and the rectosigmoid colon [1]. In gynecologic oncology, pelvic exenteration is primarily indicated for the treatment of locally advanced primary disease or isolated, centrally recurrent tumors confined to the pelvis, in the absence of distant metastases [2]. In studies, pelvic exenteration has been associated with a 5-year survival rate of 40–70%, which is substantially higher than the long-term survival observed with chemotherapy alone (generally <20%) or with supportive care without curative treatment (<5%) [3].

Pelvic exenteration was first described in 1948 by Alexander Brunschwig as a treatment for recurrent or locally advanced cervical carcinoma [4]. For descriptive and reporting purposes, pelvic exenteration can be classified as type I (supralevator), type II (infralevator), or type III, depending on the extent of the surgery. Broadly, there are three types of exenteration [5]. Anterior pelvic exenteration includes removal of the genital organs and the bladder, while posterior pelvic exenteration includes removal of the genital organs and the rectum. Total pelvic exenteration involves removal of the genital organs, bladder, and rectum. The extent of pelvic tissue and visceral resection within each is based on tumor extent and is customized to ensure complete and appropriate tumor excision [6]. Although pelvic exenteration offers locoregional disease control, its surgery is associated with high morbidity and mortality, as 30-day mortality ranges from 0.5% to 1.3%, while major complication rates are 34.5%, with up to 60% of complications being potentially life-threatening [7,8]. Patient selection is critical to optimizing the best surgical and oncologic survival outcome [9,10]. Candidates must have the potential for complete resection with negative surgical margins, adequate performance status, and sufficient cardiopulmonary and nutritional reserve to tolerate a procedure with a high risk of morbidity. Careful multidisciplinary evaluation and imaging techniques are essential to confirm the absence of disease outside the pelvis and to optimize surgical planning [11].

Pelvic exenteration results in significant loss of tissue due to the en bloc removal of the pelvic organs, creating a hollow space. This empty space with reduced underlying support results in empty pelvis syndrome. The empty space can lead to fluid accumulation and small bowel translocation into the pelvis, which in turn risks abscess formation, bowel obstruction, fistula formation, and pelvic floor prolapse. Most current series report that approximately 60–90% of patients experience at least one postoperative complication after open surgery. Reported complication profiles vary between studies and include pelvic or intra-abdominal abscess formation (4–5%), bowel obstruction or ileus, and genitourinary or enteric fistula formation. In selected open pelvic exenteration series, vesicovaginal fistula rates range from 3 to 6%, while rectovaginal fistula rates have been reported between 11 and 14%; complication rates vary depending on patient selection, prior pelvic radiotherapy, and the extent of resection [12,13,14]. Exenteration also causes delayed wound healing and functional losses. Reconstructive surgery reduces complications by filling the dead space with healthy vascularized tissue, accelerates primary healing, and improves quality of life. It also provides anatomical integrity for pelvic support. Therefore, reconstruction is a critical step in improving both oncologic and functional outcomes [14,15]. In the last few decades, reconstructive techniques following pelvic exenteration have evolved. Closure techniques initially depended on partial-thickness skin grafts or primary approximation; however, these methods were insufficient for large pelvic defects and were linked to high rates of wound complications and fistula formation. Later, pedicled muscle flaps, such as the gracilis and gluteal, were introduced to increase vascularity in irradiated areas; however, their limited volume and reach restricted effectiveness in closing large pelvic spaces [16]. The introduction of the VRAM flap in 1977 marked a significant advancement, facilitating substantial vascularization via the deep inferior epigastric system, providing adequate tissue volume to occupy the pelvic cavity, and the adaptability for concurrent neovaginal reconstruction [17,18]. These characteristics, along with the positive outcomes observed in previously irradiated patients, have established VRAM as the current gold standard and “workhorse” flap for pelvic reconstruction [19]. Though other options like the anterolateral thigh flap, perforator-based flaps, and free tissue transfers are still useful in some cases, VRAM has become the most popular method because it can fix complicated composite defects, is technically feasible, and is dependable [20].

In recent years, pelvic exenteration procedures have increasingly been performed laparoscopically and, later, with robotic technology, following the proven oncological safety of minimally invasive surgery [21]. The first documented case of laparoscopic total pelvic exenteration occurred in 2003, followed by the initial robotic-assisted total pelvic exenteration in 2009. Since that time, many surgeons have documented their limited experiences with minimally invasive pelvic exenteration. The research conducted by Matsuo et al. [22] on the national uptake of minimally invasive surgery for pelvic exenteration showed that among 1376 women who underwent the procedure, 49 (3.6%) were treated using a minimally invasive approach. Robotic-assisted procedures constituted 51.0% of the total minimally invasive cases. The slower uptake of minimally invasive surgery for pelvic exenteration, despite its theoretical benefits, may be attributed not only to oncological challenges but also to a lack of experience in minimally invasive reconstruction techniques [21,23]. The primary reason for this shift is that minimally invasive techniques offer less blood loss, less wound morbidity, better visualization, and allow for faster postoperative recovery. Robotic platforms offer 3D imaging, tremor filtration, and a wider articulation angle, significantly increasing the safety of dissection in fibrotic and radiation-affected distorted pelvic structures. As such, the robotic approach to pelvic exenteration has become a standard at many centers. The robotic approach to the RAM flap allows the surgical approach to remain minimally invasive and provides a significant advantage over open RAM [24,25]. Despite the rapid uptake of robotic surgery in the field of gynecologic oncology, evidence regarding robotic rectus muscle flap reconstruction after pelvic exenteration is extremely limited. The existing literature consists of case reports, technical descriptions, and single-center retrospective series with small patient numbers. Standardized outcome measures and comparative analysis data are lacking. Importantly, no review exists that consolidates available literature or examines robot-assisted rectus muscle-based flap reconstruction in the context of minimally invasive exenteration and reconstructive surgery practices. There is a lack of guidance on patient selection, surgical technique, perioperative expectations and management, and future research priorities. Therefore, our review aims to provide a comprehensive summary of available evidence, clarify the role of robot-assisted rectus muscle-based flap reconstruction in modern reconstruction algorithms, and propose a structured framework for oncologic surgeons to integrate this technique into pelvic exenteration surgery.

## 2. Materials and Methods

The aim of this narrative review is to summarize all studies on pelvic exenteration followed by robotic rectus abdominis reconstruction in gynecologic oncology. This narrative review was conducted using established principles for high-quality narrative synthesis, with careful attention to transparent study selection, critical appraisal, and balanced interpretation of the available literature in alignment with the SANRA criteria [26]. A comprehensive literature search was conducted in PubMed(National Library of Medicine, Bethesda, MD, USA), Embase (accessed via Ovid) (Wolters Kluwer, Alphen aan den Rijn, The Netherlands), Scopus(Elsevier, Amsterdam, The Netherlands), and Web of Science (Clarivate, London, UK) to identify all studies reporting robotic rectus abdominis muscle flap (RAM/VRAM) reconstruction performed after pelvic exenteration in gynecologic oncology. The search covered publications from January 2000 to November 2025. A structured broad-to-narrow strategy was applied in all databases.

1. Broad search for pelvic exenteration.
2. Search for VRAM/RAM/rectus abdominis flap reconstruction.
3. Search for robotic or robot-assisted surgery.
4. Combination of the three domains using the Boolean operator AND.

No restrictions on study design were applied. Eligible studies included retrospective or prospective clinical studies, technical reports, case series, and case reports involving robotic RAM/VRAM reconstruction after pelvic exenteration. Non-robotic reconstruction, animal studies, and studies not including rectus-based flaps were excluded.

## 3. Results

### 3.1. Literature Search and Study Characteristics

The database-specific counts were as follows:

PubMed: Pelvic exenteration (2223 records), rectus/VRAM/RAM flaps (551 records), robotic surgery (69,960 records). The final combined query (pelvic exenteration AND rectus flap AND robotic) identified one record.

Embase (Via Ovid): Pelvic exenteration (3389 records), rectus/VRAM/RAM flaps (845 records), robotic surgery (121,664 records). The combined search returned seven records.

Scopus: Pelvic exenteration (3183 records), rectus/VRAM/RAM flaps (72,728 records), robotic surgery (461,715 records). The combined query yielded three records.

Web of Science: Pelvic exenteration (2454 records), rectus/VRAM/RAM flaps (39,001 records), robotic surgery (166,871 records). The combined search identified three records.

**Figure 1:**
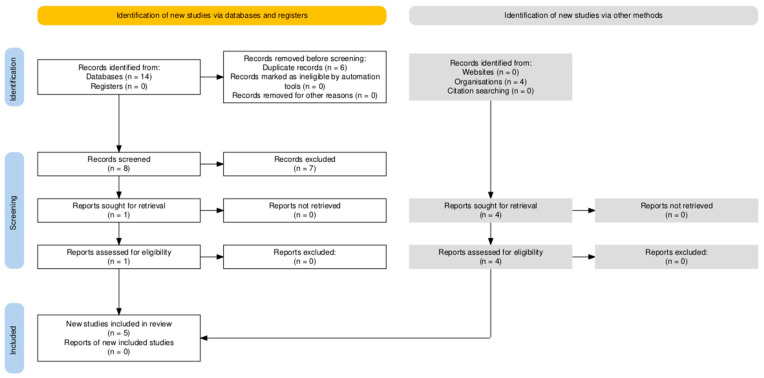
Prisma 2020 flow diagram showing the identification and selection of studies evaluating robotic rectus abdominis flap reconstruction after pelvic exenteration in gynecologic oncology.

All records from each database were exported and imported into the Zotero reference management software (version 6.0). Zotero’s “Duplicate Items” feature was used to automatically identify and merge duplicate citations. Following deduplication, a total of eight unique studies remained and were screened. After applying the primary Boolean filter (pelvic exenteration AND rectus flap AND robotic), one directly relevant record was retrieved. Because robotic rectus flap reconstruction is underreported in indexed databases, broader search sets were manually screened for additional relevant studies. This process identified a total of five studies that were included in the qualitative synthesis [27,28,29,30,31] (Figure 1).

### 3.2. Summary of Reported Outcomes (Feasibility, Complications, Recovery)

Across five studies (Pedersen, *n* = 10; Haverland, *n* = 6; Kelecy, *n* = 14; Iftekhar, *n* = 5; Singh, *n* = 3), a total of 38 robotic rectus abdominis muscle flap (RRAM) procedures were reported [27,28,29,30,31]. There were no cases of flap loss reported (0/38; 0%). Operative metrics varied among studies. Robotic harvest time ranged from 31 to 126 min (mean 45 min) in the Pedersen series [27], whereas console time reached 424–446 min in the Kelecy series [29]. The Singh series [31] reported a median total operative time of 522 min. Console and harvest times were not separately reported in the Haverland [28] and Iftekhar [30] studies. The length of hospital stay varied across the studies. Haverland [28] reported a median duration of 3 days, whereas Singth [31] reported a median of 6 days. The overall complication profile was favorable, as determined by no flap necrosis in any of the reported studies (0%) and all flaps healing well. Only four major complication events, including perineal dehiscence requiring exploratory laparotomy (*n* = 1), abscess/hematoma requiring drain (*n* = 2), and urethrocutaneous fistula (*n* = 1), were reported (both in the Kelecy series) [29]. Minor complications, such as wound breakdown, surgical site infection and parastomal hernia, were observed in approximately one-third of the cases (34–36%). Reported events included one perineal wound infection (Singh [31]), two exenteration-related complications—intermittent bowel obstruction and pyelonephritis with pelvic abscess (Haverland [28])—three non-flap related complications—one late vaginal stenosis, and one superficial perineal dehiscence (Iftekhar [30])—and one minor pressure ulcer (Pedersen [27]). Kelecy [29] reported 11 minor complications in addition to the four major events (Table 1).

## 4. Discussion

### 4.1. Pelvic Anatomy and Reconstruction Requirements

The success of pelvic exenteration in the treatment of recurrent or locally advanced gynecologic cancers depends not only on complete resection of the tumor but also on the closure of the resulting large defect. The dead space created after pelvic exenteration, impaired pelvic blood supply due to previous radiation therapy, and altered pelvic floor biomechanics increase the risk of infection and impair wound healing [32].

Therefore, the rectus abdominis muscle, supplied by the deep inferior epigastric system, provides strong axial blood flow to support tissue healing in previously radiated and fibrotic pelvic areas. The rectus abdominis muscle also provides structural support within the pelvis and shares the load so that it is not directed to the fragile perineal region, decreasing risk for prolapse or herniation of the perineal body [33].

Another important point in rectus muscle reconstruction is the preservation of abdominal wall anatomy. With the robotic intraperitoneal harvest technique, the rectus muscle is opened from the posterior sheath without touching the anterior sheath and linea alba, thus minimizing the risk of abdominal hernia formation [27]. From all these perspectives discussed above, the findings of our narrative review have shown that pelvic reconstruction is quite effective in addressing wide and complex anatomical and physiological needs in a compromised pelvic environment.

### 4.2. Flap Design and Variants (RAM (VRAM/TRAM), RAMP)

From a reconstructive standpoint, the primary goal after pelvic exenteration is the transfer of well-vascularized, non-irradiated tissue to promote neovascularization, fill pelvic dead space, and optimize functional and cosmetic outcomes while minimizing complications [34]. Among rectus-based options, VRAM is often preferred when reliable perfusion and adequate tissue volume are required, whereas alternative flaps may be limited by procedure-related or flap-specific constraints [19]. Gluteal flaps may be suboptimal when gluteal vessels are sacrificed during extensive pelvic resection, and gracilis flaps, although useful when VRAM is not feasible, are more delicate and susceptible to pedicle injury during rotation [35]. Importantly, VRAM reconstruction has limitations and may be less suitable in cases requiring bilateral ostomies, in the presence of prior transverse or Maylard incisions, or after previous abdominal surgery where the inferior epigastric vessels may have been compromised; these factors should be carefully considered during preoperative planning [36]. Collectively, these considerations define the key indications and relative contraindications for VRAM-based reconstruction that demonstrate the value of individualized flap selection in pelvic exenteration. Rectus-based flaps represent the cornerstone of pelvic reconstruction after exenteration, but they differ in design and functional outcomes. RAM flaps generally include vertical (VRAM) and transverse (TRAM) orientations and consist of subcutaneous tissue and muscle, while myoperitoneal flaps (RAMP) incorporate peritoneum rather than muscle and skin. The traditional VRAM flap has long been established as the standard option for open pelvic reconstruction. Berger J et al. [18] in their review and meta-analysis, evaluated 46 pelvic exenteration cases in terms of modified-vertical rectus abdominis myocutaneous flap usage and reported that flap-specific morbidity was 19.6% (*n*: 9 patients, but complete and superficial flap necrosis was three cases); vaginal stenosis, 6.5% (*n*: 3 cases); pelvic abscess, 30.4% (*n*: 14 cases); and anterior abdominal wound separation, 47.8% (*n*: 22 cases). In this modification method, they used a smaller conical skin paddle, which reduces the donor’s fascial defect and often eliminates the need for mesh. They also highlighted that prior transverse/Maylard incisions may jeopardize the pedicle. It is important to remember that 82.6% of the patients in this study were high-risk. Because high-risk factors such as obesity, poor nutritional status, diabetes mellitus, smoking, advanced age, previous surgery, and radiation history can increase the complication rate, as in this case, we believe that minimal invasive surgery should be preferred whenever possible.

In two separate studies conducted one year apart by Soper et al. [37], they compared neovaginal reconstruction cases that underwent radical pelvic surgery followed by vertical RAM and transverse RAM, and no significant difference was observed between them in terms of flap-specific complications. In their second study, they compared 32 cases undergoing RAM with 7 cases undergoing RAMP and found that 43% of the RAMP group had complete vaginal stenosis, while this rate was only 3% in the RAM group. Based on this result, they emphasized that using the peritoneum instead of skin in RAMP flaps was effective for partial vaginal defects, but in cases with large circumferential vaginal defects, such as those involving more than 65% of the vagina, 100% stenosis was observed and was unsuccessful. However, in cases undergoing RAM, the stenosis rate was zero [38]. These findings underscore the critical role of tissue composition in vaginal reconstruction. The high rates of stenosis observed with peritoneal or muscle-only flaps highlight the limitations of approaches lacking a cutaneous component, particularly in large or circumferential vaginal defects. The reconstructive value of a VRAM flap extends beyond technical feasibility, largely owing to the substantial volume of skin and adipose tissue it provides. This tissue bulk plays a critical role in effective obliteration of pelvic dead space, in preventing descent of irradiated bowel into the pelvis, and in reducing the risk of secondary perineal herniation, an often-challenging complication to manage. From a reconstructive perspective, these attributes warrant greater emphasis. Accordingly, although robotic harvest is technically feasible, feasibility alone should not be interpreted as reconstructive equivalence to a classical transpelvic VRAM flap. A related technical consideration that merits explicit attention is the method of flap fixation. Securing a muscle-only rectus flap to the pelvic floor can be challenging, as muscle tissue alone does not tolerate high-tension suturing and is consequently vulnerable to tearing or ischemic compromise. Durable fixation and long-term positional stability typically rely on the presence of fascial, cutaneous, or peritoneal elements, which provide more robust structural support. In their absence there is a reasonable concern that a mobilized muscle flap may not remain adequately positioned within the pelvis over time [39,40]. Rectus abdominis flap reconstruction after pelvic exenteration carries a substantial risk of postoperative wound complications, with reported rates ranging from 15 to 35%; issues include wound dehiscence, infection, partial flap necrosis, and, rarely, flap loss. Several adjunctive techniques such as extended flaps, vacuum-assisted drainage, and omental augmentation have been shown to reduce these complications, with omental flaps in particular demonstrating lower pelvic infection rates. Effective management ultimately relies on obliterating the pelvic dead space, a key step in preventing major postoperative problems such as bowel obstruction and peritonitis [41].

In a study conducted by Cibula et al. [39], patients who underwent modified rectus abdominis myoperitoneal (RAMP) flap surgery for pelvic floor reconstruction (*n* = 16) were compared with a historical control group (*n* = 24) who underwent pelvic exenteration without reconstruction. Both groups were similar in terms of demographic and tumoral characteristics, but both early and late postoperative complications were significantly less in the RAMP group. Furthermore, incisional hernia formation was also observed less frequently in the RAMP group. In this study, Cibula et al. [39] demonstrated that the RAMP flap promotes functional recovery by reducing complications related to empty pelvis syndrome when compared with the pelvic exenteration without reconstruction group. Another study by Cortinovis et al. [42] observed that the rectus abdominis myofascial flap (RAMF), consisting of the rectus muscle and its anterior fascia without skin, effectively fulfills both anatomic and functional reconstruction needs following pelvic exenteration. The absence of skin within the RAMF eliminated problems such as hair follicles and associated sebum production, thus significantly facilitating the gradual mucolization process that occurs over a period of 40–60 days. In the same study, Cortinovis et al. [42] found no flap necrosis in their series of 16 pelvic exenteration cases, and only 12.5% had flap-related complications. They observed significant improvements in both the quality of life (SF 36) and sexual function scores (FSFI) of patients over a 12-month period. Building on the proven anatomical and functional reliability of RAM and RAMFs in open surgery, emerging evidence from our multiple-robotic-series review demonstrates that robotic-assisted approaches are not only feasible and safe but also maintain low morbidity, excellent donor-site outcomes, and successful reconstruction without flap loss. These findings support the idea that robotic platforms improve visualization, ergonomics, and placement accuracy in complex pelvic reconstructions [43]. It is important to clearly distinguish robot-assisted rectus muscle-based flap reconstruction from a true transpelvic vertical rectus abdominis myocutaneous (VRAM) flap. The majority of published robotic experiences involve muscle-only or myoperitoneal variants that lack a skin paddle and are therefore not functionally equivalent to a myocutaneous VRAM flap. This distinction is particularly relevant in cases requiring extensive vaginal wall resection and reliable pelvic floor reconstruction, where the absence of a cutaneous component limits the ability of muscle-only robotic approaches to reconstruct large or circumferential vaginal defects. However, this limitation does not imply that minimally invasive oncologic pelvic surgery must rely on muscle-only reconstructions. Earlier hybrid techniques reinforce that minimally invasive pelvic oncologic surgery can be effectively combined with optimized VRAM reconstruction. Horch et al. [44] demonstrated this by pairing laparoscopic abdominoperineal resection with a minimized-access transpelvic VRAM flap, showing that modern minimally invasive extirpation, including robotic approaches, remains compatible with the functional advantages of a true myocutaneous VRAM rather than muscle-only substitutes. Their technique showed that a composite flap containing fascia, a skin paddle, and subcutaneous tissue can maintain stable pelvic positioning without mesh reinforcement, underscoring that long-term stability largely derives from these structural components. This hybrid experience provides a clear example that minimally invasive surgery and durable VRAM-based reconstruction are not mutually exclusive.

### 4.3. Robotic Technique: Advantages, Ergonomics, and Feasibility

Robotically assisted rectus abdominis muscle flap harvesting and subsequent reconstruction after challenging pelvic surgeries for pelvic reconstruction provides significant technical and ergonomic advantages. Hammond et al. [45] reported that intraperitoneal, sheath-sparing posterior rectus dissection performed through existing minimally invasive ports avoids laparotomy or additional midline incisions and preserves the anterior rectus sheath. In their study, İbrahim et al. [46] emphasized the three-dimensional visual feature of the platform and the ergonomic advantages of the wrist movement instruments; they stated that these features increased the surgeon’s visual depth and skill, allowing the flap to be easily placed by precisely elevating it in the limited pelvic area.

In addition to the above studies, Davila et al. [47] reported in another study that coordinated workflows enabled the robot to complete the resection and reconstruction processes by integrating them seamlessly. In the same study, it was also stated that by removing the posterior rectus sheath and adding a mesh, when necessary, less swelling and hernia formation were observed in the abdominal wall after surgery. Based on feasibility results from early clinical series in the literature, Haverland et al. [28] documented successful pelvic wound healing in a heterogeneous cohort, including pelvic exenteration and fistula repair, with only one readmission and no harvest-related conversion. In another study, Asaad et al. [48] compared their robotic sheath-sparing harvesting method with the open technique, which has been widely used in the past. In their comparative analysis of perineal constriction, they observed that rectus muscle removal with the robotic method resulted in shorter hospital stays and fewer minor complications compared to the traditional open method.

Particularly in the context of pelvic exenteration, Winters et al. [49] described their experience with three patients who had robotic total pelvic exenteration with laparoscopic rectus flap and compared them with their open surgeries. They reported lower blood loss, shorter intensive care unit stay, and reduced overall length of hospital stay, but similar operation times in their robotic cases. The findings from our narrative review are consistent, significant, and aligned with the existing literature studies on robotic technique above.

The findings from our narrative review are highly consistent with the existing literature on robotic techniques. The high flap survival, low major complication rates, and minimal donor site morbidity we report align with the technical advantages of preserving the anterior sheath described by Hammond et al. [45] and Ibrahim et al. [46], and with Asaad et al.’s [48] data demonstrating a significant reduction in abdominal wall morbidity compared to open surgery. The robust wound healing and absence of flap loss are attributed to the high-quality 3D imaging provided by robotic platforms and the technical advantages offered by wrist-jointed instruments [43]. Similarly, the lower blood loss and shorter intensive care and hospital stays reported in Winters et al.’s [49] study comparing robotic and open pelvic exenterations support the preservation of minimally invasive surgical flow during the reconstruction phase that was align with our narrative review.

### 4.4. Technical Challenges and Learning Curve

While robotic rectus abdominis flap harvesting for pelvic reconstruction is technically feasible, certain points must be considered. Hammond et al. [45] and Pederson et al. [27], in their studies, stated that intraperitoneal dissection should be performed along the posterior rectus sheath, meticulously protecting the inferior epigastric pedicle, while the anterior sheath should be intentionally left intact to prevent future hernia development. They noted that this often requires reorientation of the robot toward the posterior abdominal wall, additional lateral mobilization for access, and the need for console handoffs between the extirpative and reconstructive teams, all of which complicate the workflow. Pedersen et al. [27] and Singh et al. [31] claimed that early workflow metrics comprise approximately 15 min for robotic setup in experienced teams and an average of 45 min for intraperitoneal rectus flap harvest using three ports. However, when oncologic and reconstructive procedures are combined, the time can be significantly longer; for example, a median operative time of 522 min was reported in a series of tandem robotic ELAPE rectus flap surgeries, highlighting the time loss associated with redocking and field changes [31]. Learning-curve data from robotic abdominal flap surgery indicates that the ability to perform pedicle dissection increases significantly after the first five to ten cases, becoming more standardized and efficient [50]. Nevertheless, because reconstruction is often performed after complex pelvic oncology surgeries, such as for pelvic fibrosis and anatomic distortions, and requires the integration of multidisciplinary teams into surgeries, a longer learning curve is required. Therefore, gradual adoption, structured supervision, and coordinated multidisciplinary planning are recommended during early program implementation [51,52].

The robot-assisted rectus muscle-based flap reconstruction is presently in the Development Phase (Stage 2a) of the IDEAL framework, marked by initial experiences (generally fewer than 30 cases) and ongoing technical adjustments. Future reporting should evolve from basic feasibility studies to prospective, single-center case series that employ standardized flowcharts for documenting technical modifications and early failures. Stability of the technique is a prerequisite for transitioning to the Exploration Phase (Stage 2b), during which multicenter cohort studies will be essential to delineate the learning curve and establish the baseline data required for subsequent randomized assessments [53].

## 5. Future Directions

To advance in robotic abdominis muscle flap reconstruction, it is necessary to move beyond the feasibility data reported in existing studies towards multicenter prospective studies, which can assess long-term oncologic equivalence with open surgery. Although early series demonstrate perioperative advantages such as reduced blood loss and shorter hospital stays, current evidence of recurrence and survival is insufficient, underscoring the need for standardized protocols [49]. From a technical perspective, robotic rectus muscle flap preparation may reduce abdominal wall morbidity by preserving the anterior rectus sheath; however, the effect of strategies such as prophylactic mesh use and posterior sheath dissection on donor site hernia is uncertain, and comparative studies are needed. While minimally invasive exenteration is reported to be associated with reduced complications and lower costs, recent data indicates that vaginal reconstruction rates remain low. Therefore, this low rate shows that reconstruction is not being performed adequately during robotic pelvic exenteration and highlights the need for standardization of port placement, robotic configuration, and team coordination. Standards are also needed for flap selection; current evidence suggests that RAMP is not suitable for large vaginal defects due to high stenosis rates, and VRAM/RAM should be preferred [47,48,54].However, the learning curve for robotic technique has not been defined; initial analog data suggests that technical proficiency can be achieved in approximately 10 cases, so the development of training and simulation-based programs is crucial [50]. Finally, there is a significant gap in long-term patient outcomes. For example, although persistent problems in sexual function, body image, and pelvic function have been reported despite VRAM reconstruction, future studies should objectively assess these outcomes using standardized measurement tools (e.g., FSFI, EORTC QLQ) and support reconstruction planning with advanced imaging modalities [55,56]. While robot-assisted rectus abdominis flap reconstruction preserves the minimally invasive advantages of robotic pelvic exenteration, evidence regarding its cost-effectiveness compared to open or conventional laparoscopic reconstruction is limited. When evaluating robot-assisted surgery from an economic perspective, studies report heterogeneous and often inconclusive results, influenced by variability in healthcare settings, costing methodologies, and platform-specific factors such as capital costs and utilization. Furthermore, access to robotic platforms is largely limited to high-income and developed countries. Importantly, the learning curve associated with robotic platform use should be distinguished from the learning curve of individual surgical procedures; current evidence suggests that platform proficiency constitutes the primary component of the learning curve, while procedure-specific reconstructive steps are learned gradually after gaining robotic experience [57].

In the future, artificial intelligence will hold promise for personalized planning and management in robotic RA flap reconstruction after pelvic exenteration by enabling deep learning-based CTA analysis (automatically mapping the deep inferior epigastric system and perforator trajectories with submillimeter accuracy and near-real-time runtimes), intraoperative decision support through AI-driven augmented reality (AR) and mixed reality (MR) platforms, indocyanine green based (ICG) protocols, early complication detection with hyperspectral imaging, and machine learning-based risk prediction [58,59,60,61]. However, the heterogeneity of surgical techniques and the limited number of reported cases also constrain the strength of the clinical recommendations that can be drawn. In addition, the predominantly short-term follow-up in the available studies limits the ability to adequately assess late complications, particularly perineal hernia, which may manifest several months or years after pelvic exenteration.

## 6. Conclusions

Robotic rectus abdominis flap reconstruction is emerging as a significant advancement in the reconstructive management of patients undergoing pelvic exenteration for advanced or recurrent gynecologic cancers. Although current evidence is limited, it consistently indicates that robotic flap harvesting is technically feasible and associated with excellent flap viability, minimal donor-site morbidity, and faster postoperative recovery. However, long-term oncologic equivalence has not yet been established, and no oncologic safety concerns have been reported to date. Nevertheless, evidence on robot-assisted rectus muscle-based flap reconstruction after pelvic exenteration remains limited to small series and case reports, making it susceptible to selection and publication bias. Variability in surgical technique and inconsistent outcome reporting precluded quantitative pooling. Larger, comparative multicenter studies with standardized endpoints are needed to clarify patient selection, reproducibility, and long-term outcomes. Although these results are promising, the current literature is still not complete and lacks standardized ways to report surgical criteria, complication profiles, long-term pelvic and sexual function, and comparative outcomes with open VRAM or alternative flap designs. The lack of high-quality prospective or multicenter studies precludes definitive conclusions regarding the generalizability and long-term benefits of robotic reconstruction. Therefore, the development of structured protocols, the adoption of common outcome measures, and the establishment of collaborative, multicenter registration systems are critical to determining the true status of robot-assisted rectus muscle-based flap reconstruction in modern reconstructive algorithms. As minimally invasive pelvic exenteration becomes more common in gynecologic oncology, reconstructive strategies should adapt in parallel. Robot-assisted rectus muscle-based flap reconstruction has emerged as an essential part of these workflows, helping lower wound-related complications and support better functional recovery when applied to carefully selected patients using standardized methods. Future technologies, such as AI-powered imaging, augmented reality-guided dissection, and predictive algorithms, are poised to improve the accuracy, safety, and individualized planning of robotic pelvic reconstruction. Ultimately, the successful adoption of robotic rectus flap reconstruction will rely on continued technological progress, specialized training programs, and robust clinical research aimed at enhancing both surgical outcomes and patient quality of life in these highly complex gynecologic oncology procedures.

## Figures and Tables

**Table 1:** Summary of published studies evaluating robotic rectus abdominis muscle reconstruction.

Author (Year)/Reconstruction Method Used	Study Type	Patients (*n*)	Used Robotic Platform	Console Time/Operation Time	Complications (*n*)	Length of Hospital Stay	Key Outcomes
Haverland et al., [28] (2020)/Muscle-only rectus flap (robot-assisted)	Single-Institution Experience, Case Series	6	Da Vinci Surgical System (Intuitive Surgical, Sunnyvale, CA, USA)	Not mentioned	*n*: 2 * Intermittent bowel obstruction * Pyelonephritis from ileal conduit with pelvic abscess infection Note: * 1 conversion to laparotomy (non-harvest-related)	3 days (1–6)	This technique proved feasible and safe, with no flap loss, low morbidity, good wound healing, and only limited complications, all while demonstrating successful reconstruction without harvest-related issues.
Singh et al. [31] (2015)/Muscle-only rectus abdominis flap (robot-assisted)	Retrospective Pilot Study (* Note: gynecologic oncology; colorectal ELAPE case series, but technique transferable to pelvic exenteration reconstruction.)	3	Da Vinci Surgical System (Intuitive Surgical, Sunnyvale, CA, USA)	Median 522 min	1 * 1 perineal wound complication reported * No hernia	6 days (6–9 days)	Feasible, safe robotic RAM harvest with successful pelvic floor closure.
Kelecy et al. [29] (2025)/Muscle-only rectus flap (robot-assisted)	Single-Institution Retrospective Review	14	Da Vinci Surgical System (Intuitive Surgical, Sunnyvale, CA, USA)	* 424 (92) min for two surgeons * 446 (57) min for three surgeons	11 minor, 4 major * Minor complications: 5 cases for 2 surgeons * Minor complications: 6 cases for 3 surgeons * Major complications were 2 cases each for 2 and 3 surgeons	* 8 (2.1) days (2-surgeon) * 12 (12) days (3-surgeon)	Adding a third surgeon did NOT reduce operative time, console time, LOS, or complication rates. Plastic surgeons are capable and should familiarize themselves with robotic technology
Iftekhar et al. [30] (2025)/Rectus abdominis myoperitoneal flap (robot-assisted)	Case Review	5	Da Vinci Surgical System (Intuitive Surgical, Sunnyvale, CA, USA)	Not reported	Vaginal stenosis: 1 case Minor wound complications amenable to topical treatment:1 case	There were no readmissions, and all patients demonstrated successful early postoperative healing.	No major complications reported. Return to OR or Readmissions: not reported Only minor, manageable issues; universal successful healing.
Pedersen et al. [27] (2014) Muscle-only rectus abdominis flap(robot-assisted)	Technical Description/Multi-Institutional Case Report	*n*: 10	Da Vinci Surgical System (Intuitive Surgical, Sunnyvale, CA, USA)	Robotic harvest time: mean 45 min (31–126 min)	1 No major complications, one minor complication (Stage one decubitus ulcer)	Not reported	Safe, efficient, and reproducible technique with no conversions, no flap loss, minimal morbidity, and excellent donor-site outcomes

Note: * Indicates reported complications.

## Data Availability

All data supporting the findings are available within the article and its tables. No additional datasets were generated.
